# Collision-based energetic comparison of rolling and hopping over obstacles

**DOI:** 10.1371/journal.pone.0194375

**Published:** 2018-03-14

**Authors:** Fabio Giardina, Fumiya Iida

**Affiliations:** Department of Engineering, The University of Cambridge, Cambridge, United Kingdom; Universitat Zurich, SWITZERLAND

## Abstract

Locomotion of machines and robots operating in rough terrain is strongly influenced by the mechanics of the ground-machine interactions. A rolling wheel in terrain with obstacles is subject to collisional energy losses, which is governed by mechanics comparable to hopping or walking locomotion. Here we investigate the energetic cost associated with overcoming an obstacle for rolling and hopping locomotion, using a simple mechanics model. The model considers collision-based interactions with the ground and the obstacle, without frictional losses, and we quantify, analyse, and compare the sources of energetic costs for three locomotion strategies. Our results show that the energetic advantages of the locomotion strategies are uniquely defined given the moment of inertia and the Froude number associated with the system. We find that hopping outperforms rolling at larger Froude numbers and vice versa. The analysis is further extended for a comparative study with animals. By applying size and inertial properties through an allometric scaling law of hopping and trotting animals to our models, we found that the conditions at which hopping becomes energetically advantageous to rolling roughly corresponds to animals’ preferred gait transition speeds. The energetic collision losses as predicted by the model are largely verified experimentally.

## Introduction

Rolling wheels are designed to operate in flat environments and are optimised for this specific domain. The economic costs for transportation drive the design of wheeled vehicles towards ever less fuel consumption, defining energy expenditure as one of the main objectives to be minimised. While a rolling strategy is undoubtedly dominating flat terrains in terms of energy expenditure, the strategy should be reconsidered in more complex environments. Wheeled locomotion in natural terrain has been extensively studied in the field of terramechanics [1], which employs empirical and computational tools to model and predict the soil-vehicle interaction. Terramechanics has aided the design of off-road vehicles ever since cars left roads, and with the advent of planetary exploration rovers, which need to operate in surroundings with loose soil and variable terrain conditions [2], more challenging terrain-wheel interactions are being analysed.

Alternatives to rolling are readily displayed by nature’s crawling, hopping and running animals, but can these gaits compete with the energy effective rolling motion? In a study where cyclists compete against runners in an off-road track, it was found that their energy expenditure is comparable [3], but cyclists finished significantly faster. This implies that even when rolling and running strategies are compared on an equal footing, the rolling strategy still outperforms the running one in terms of its speed. This comparison, however, is commonly done in environments which lack an important element of natural terrain: obstacles. The energetic cost of a wheel colliding with obstacles is a classical problem in mechanics [4], yet it is generally only studied for rolling collisions.

The study of the rimless wheel [5–6] calls attention to the similarities of rolling and walking collisions, and links wheeled locomotion to legged locomotion. Collision mechanics is a commonly used tool for analysis of legged locomotion, as repetitive leg-ground interactions are typical in this form of locomotion. The simplest models reduce the analysed system to a single body with point mass and look at the momentum balance of the collision such as in [7], where the authors explain observed locomotion behaviour in the walk to run transition, and the elastic behaviour in stance phase during running. An analogous approach studies collisional behaviour of quadrupedal animals [8] and shows that walk and gallop provide collision reduction strategies in stance phase. Collisional analysis of gaits is not confined to legged locomotion, but has also been used to study arboreal locomotion, such as brachiation in gibbons, to explain the observed overshoot during swinging motion [9]. Other collision-based models study two-bodied locomotion, such as in [10] where toe-off impulses acting on a two-legged system are investigated. A similar system is introduced in [11], where the concept of passive dynamic walking is studied in a collisional context. Extensions of the two-legged models also investigate the effect of mass distribution on stability as described in [12] and [13]. Collision-based models have not only led to the advancement of our theoretical understanding of locomotion, but also facilitated the creation of dynamic walking robots [14]. Even though collision-based models have been studied extensively in the past, the effect of mass distribution on the energetic cost of impulsive events have not been investigated in depth, which we deem crucial for the study of a rotating and hopping wheel.

In this work, we make use of the collision-based modelling approach to analyse energy expenditure of three strategies in a wheeled system in the task of overcoming an obstacle, and explicitly emphasise the role of mass distribution in the energetic analysis of impulsive events. We quantify mass distribution by the wheel’s moment of inertia around the centre of mass, which uniquely defines the inertial properties of the rigid body. We find a new way of overcoming the obstacle which is associated with low energetic costs in the case of a small moment of inertia. The model thus underscores the importance of moment of inertia and centre of mass position in locomotion with collisional events and suggests a new perspective to understand and induce hopping. Other than the quantitative analysis of energetic advantage of hopping and rolling, this model can provide useful insights to the study of moment of inertia-dependent dynamics in locomotion such as in swing-leg retraction [15], posture control during flight phase [16], and balance during stance phase [17].

**Fig 1:**
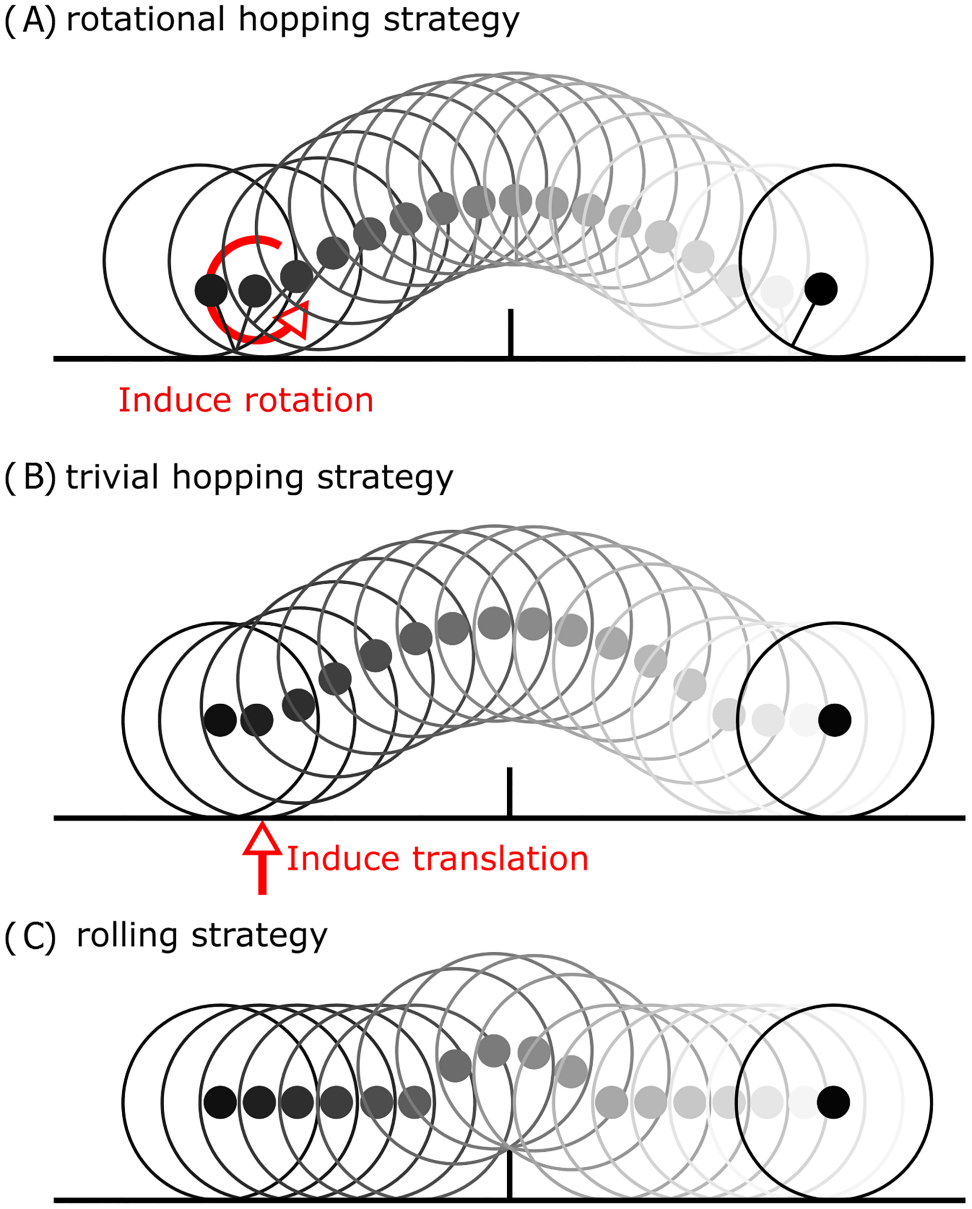
Locomotion strategies. The motion progression of the three studied strategies to overcome an obstacle. Energetic cost of collision is derived and completely defined for each strategy by wheel radius *R*, eccentricity *a* of the wheel, the mass located in the centre of mass *m*, the moment of inertia around the centre of mass *I*, the obstacle height *h*, and the approach velocity *u*_*x*_. (A) Motion progression of the rotational hopping strategy using an off-centred wheel. Hopping is induced by reverting the rotation during stance phase. (B) Motion progression of the trivial hopping strategy using a wheel with centred mass. Hopping is induced by an increase of the kinetic energy in vertical direction. (C) Motion progression of the rolling strategy. The obstacle is overcome by a wheel with centred mass colliding and rolling over it.

We test and compare the energetic cost of three distinct strategies for a rigid wheel to overcome an acute and rigid obstacle of given height, as illustrated in Fig 1. We use the result of this comparison to find conditions for which hopping is energetically advantageous to rolling. We then take the theoretical result and use it to study its prediction on animal-related locomotion conditions. The three strategies we analyse are governed by different physical effects to overcome the obstacle. The rolling strategy shown in Fig 1C is characterised by a wheel with centred mass colliding with the obstacle and subsequently rolling over it. In the trivial hopping strategy as shown in Fig 1B, the kinetic energy of the system is increased in vertical direction by the potential energy required to overcome the obstacle (*E*_*TH*_ = *mgh*). We introduce a third strategy with the rotational hopping strategy as shown in Fig 1A. This strategy exploits rotation to overcome the obstacle and–as we will see–not only surpasses the trivial hopping strategy in terms of energy effectiveness, but also displays characteristics of legged hopping locomotion. The wheel’s mass distribution in this strategy is such that the centre of mass is off-centred, due to which the centre of mass velocity will eventually have a component in vertical direction as the wheel rolls over the ground. At a point where the velocity is directed away from the ground, an induced rotation causes the wheel to revert its angular velocity. This forces the boundary of the wheel to move away from the ground, which thus leads to a change from rolling to a ballistic flight phase. The cost of this strategy is composed of the energetic cost of reverting the rotation at take-off and the energetic cost of collision at touchdown.

The model for all strategies is completely described with its wheel radius *R*, its eccentricity *a* (for the rotational hopping strategy), the mass located in the centre of mass *m*, the moment of inertia around the centre of mass *I*, the obstacle height *h*, and the approach velocity *u*_*x*_. We will first analyse locomotion strategies without specifying mass-size relationships before we apply an allometric scaling law derived from hopping, running and trotting animals. This relates mass, radius and moment of inertia to animal properties, and invokes the question of whether animals would prefer to roll or hop in given environmental conditions.

The next section gives a detailed description of the used model and methods, followed by the results of the strategy comparison and the study of the theoretical model for allometric (animal related) scaling laws. We then verify the energetic collision losses of rolling and hopping in an experimental setup of a wheel-obstacle test platform, before we discuss the results of the strategy comparison.

## Methods

### Model assumptions

The subsequent theoretical analysis uses standard assumptions of planar rigid body mechanics. The body inertia is given by the centre of mass with the point mass *m*, and the mass distribution represented by the moment of inertia *I* around the centre of mass. Collision of the rigid body with the environment takes place at its boundary, which is a circular shape in all cases. Note that forces acting on the boundary not only accelerate the centre of mass, but, depending on their direction and point of attack, also induce a moment around the centre of mass according to the moment of inertia *I*.We model environment interactions by inelastic collisions as shown in [18] p.100, for example. This relates the pre-collision generalized velocity ***u***^−^ to the post-collision velocity ***u***^+^ by

$$u^{+}\;=\;(I-M^{-1}J^{T}{\left(JM^{-1}J^{T}\right)}^{-1}J)u^{-},$$ (1)

with **I** the identity matrix, **M** the generalised mass matrix, and **J** the Jacobian of the contact point. Note that (1) does not allow for slippage between the wheel and the environment, meaning that we do not have to model energetic losses due to friction. We will make extensive use of what we call the collision matrix **M**_C_, which maps the pre- collision generalised velocities to the dissipated energy by

$$\Delta E\;=\;\frac{1}{2}{\left(u^{-}\right)}^{T}M_{C}u^{-},$$ (2)

with

$$M_{C}≔-J^{T}{\left(JM^{-1}J^{T}\right)}^{-1}J.$$ (3)

Note that the collision matrix is simply derived by reformulating the difference in kinetic energy before and after collision, i.e. Δ*E* = 1/2(***u***^+^)^***T***^**M*u***^+^ − 1/2(***u***^−^)^***T***^**M*u***^−^, by using (1).

### Rotational hopping strategy

**Fig 2:**
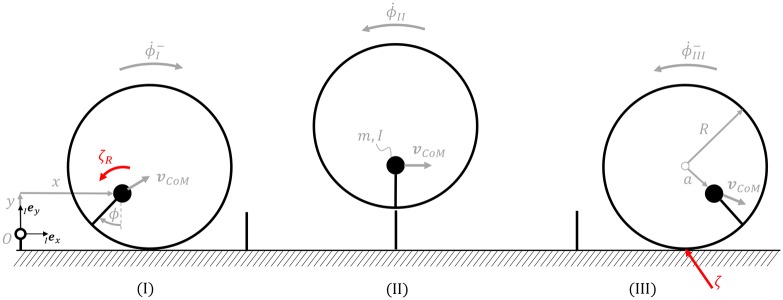
Rotational hopping model. Generalised coordinates ***q*** = [*x*, *y*, *ϕ*]^*T*^ with *x* the horizontal displacement from origin *O*, *y* vertical displacement from origin *O*, angular position *ϕ*, and system parameters mass *m*, moment of inertia *I*, wheel radius *R*, eccentricity *a*, and obstacle height *h*. (I) State just before angular impulse. The angular velocity of the system is reverted in a collisional event with angular impulse *ζ*_*R*_ from ${\dot{\phi }}_{I}^{-}$ to ${\dot{\phi }}_{II}^{\;}$. The velocity ***v***_*CoM*_ dictated by the rolling motion then leads to a ballistic flight phase. (II) Ballistic flight phase. (III) State just before impulsive energy loss due to impact *ζ*. Note that ${\dot{\phi }}_{II}^{\;}\;=\;{\dot{\phi }}_{III}^{-}$.

Fig 2 shows the off-centred wheel model for the rotational hopping strategy with the generalized coordinates ***q*** = [*x*; *y*; *ϕ*]^*T*^ with respect to an inertial frame of reference, with *x* the horizontal position of the centre of mass, *y* the vertical position of the centre of mass, and *ϕ* the angular position of the body. The radius of the wheel is denoted by the parameter *R* and its eccentricity by *a*. Note that for all the calculations relating to the rotational hopping strategy, the eccentricity was set to half the radius *a* = 0.5*R*. The ground contact Jacobian (see e.g. [18], p.23 for the definition of Jacobians) is then

$$J_{H}\;=\;\left[\begin{array}{ccc} 1 & 0 & R-a\;\;\text{cos}\;\phi \\ 0 & 1 & -a\;\text{sin}\;\phi \end{array}\right],$$ (4)

and the generalized mass matrix

$$M\;=\;\left[\begin{array}{ccc} m & 0 & 0\\ 0 & m & 0\\ 0 & 0 & I \end{array}\right].$$ (5)

We now describe the mode of locomotion of this system as also shown in Fig 2. Given a forward speed *u*_*x*_ of our system at an angle *ϕ*, the generalized pre-take-off velocity, defined through the rolling kinematics, is

uTO-=k(acosϕ-Rasinϕ1), (6)

where *k* = *u*_*x*_/(*a* cos *ϕ* − *R*) and the subscript TO indicates the take-off state. Fig 2(I) indicates that the centre of mass velocity ***v***_*CoM*_ during rolling can point away from the ground, which we exploit for a ballistic flight phase. A flight phase is induced if the angular velocity is reverted in its direction in an instant, as this interrupts the rolling motion and causes the boundary of the robot to move away from the ground rather than staying on it. We induce this change by in impulsive angular event, gauged such that the wheel reaches the angle *ϕ* = 0 at apex point. This condition is fulfilled, if the angular speed after impulse is exactly ${\dot{\phi }}^{+}\;=\;-\phi^{*}/t_{A}$, where *t*_*A*_ is the flight time to apex and ϕ* is the take-off angle. The angular impulse does not influence the translational speed of the centre of mass at take-off, which means that a ballistic motion of the centre of mass with initial velocity as given in (6) provides $t_{A}\;=\;u_{TO,y}^{-}/g$. We now find an expression for the energetic cost to change the angular speed from pre-take-off to post-take-off speed by

$$\Delta E_{R}\;=\;\frac{I}{2}\left({\left({\dot{\phi }}^{-}\right)}^{2}+{\left({\dot{\phi }}^{+}\right)}^{2}\right)\;=\;\frac{I}{2}\left({\left(\frac{\phi^{*}g}{u_{TO,y}^{-}}\right)}^{2}+k^{2}\right).$$ (7)

The first term in the above Equation is the cost to induce the required angular speed during flight phase, while the second term accounts for the braking energy required to stop the rolling motion. The impulsive actuation causes the wheel to hop over the obstacle without colliding with it. The only dissipative collision is the ground collision at touchdown. Since the ground is assumed to be flat and the flight phase symmetric, we have for the touchdown angle *ϕ*_*TD*_ = −ϕ*, and the generalised velocity at touchdown

uTD-=(k(acosϕ-R)-kasinϕ-ϕ*guTO,y-), (8)

in which we find the translational velocities through the ballistic flight phase, and the angular velocity by the requirement of a symmetric flight phase. Note the subscript TD which indicates the touchdown state. The energy loss at ground collision Δ*E*_*C*_ is then calculated using (2), where **J**_*H*_(*ϕ* = −ϕ*) is used for the Jacobian in (3) and $u_{TD}^{-}$ for the generalised velocity. The total energy consumed to overcome the obstacle is the sum of energy required to induce the backward rotation at take-off plus any energy deficit of the final energy after touchdown as compared to the initial energy. We therefore write for the total energy consumed to overcome the obstacle with the rotational hopping strategy

$$\Delta E_{H}\;=\;r(E_{0}-E_{1})+\Delta E_{R},$$ (9)

With *r*(.) the ramp function, $E_{0}\;=\;1/2({u_{TO}^{-})}^{T}Mu_{TO}^{-},$ and

$${\text{E}}_{1}\;=\;\frac{1}{2}{\left(u_{TD}^{-}\right)}^{T}Mu_{TD}^{-}+\frac{1}{2}{\left(u_{TD}^{-}\right)}^{T}M_{C}(-\phi^{*})u_{TD}^{-}.$$ (10)

Note that the argument of the collision matrix is the landing angle −ϕ*, i.e. **M**_C_(−ϕ*)**.** In our analysis, we will require the wheel to overcome an obstacle of height *h*. This will influence the required take-off angle of the rotational hopping strategy. The obstacle will be surpassed if the following implicit equation, obtained through the ballistic dynamics, is true

$$0\;=\;2g(1-a)\text{cos}\;\phi +{\left(k\;a\;\text{sin}\;\phi \right)}^{2}-2gh.$$ (11)

The solution will lead to the take-off angle ϕ* leading to a symmetric hopping strategy which surpasses the obstacle.

Note that we assume the wheel is placed at take-off such that it is exactly above the obstacle at apex point. This placement can lead to cases where the boundary of the wheel overlaps with the obstacle at take-off in the case of slow locomotion speeds. For the sake of simplicity, we do not model these interactions, and we assume that the obstacle has no effect on the hopping process.

### Rolling strategy

The collision of a rigid wheel with an obstacle is a classic problem in collision mechanics. We use the generalised rolling velocity before impact ***u***_*R*_ = *u*_*x*_ ⋅ [1,0,−1/*R*]^*T*^ which corresponds to a wheel with centred mass rolling on flat ground. The energy loss at collision can be obtained from the collision matrix with the Jacobian of the collision point

$$J_{R}\;=\;\left[\begin{array}{ccc} 1 & 0 & R-h\\ 0 & 1 & -\sqrt{2Rh-h^{2}} \end{array}\right].$$ (12)

We assume the coefficient of restitution to be negligible, thus allowing for inelastic collisions. We derive the collision loss using (2) with Jacobian as in (12)

$$\Delta E_{RC1}\;=\;\frac{1}{2}u_{R}^{T}{\text{M}}_{C}u_{R}\;=\;\frac{mu_{x}^{2}}{2}\cdot \;\frac{h(2mR^{2}-Rhm+2I)}{R(mR^{2}+I)}.$$ (13)

The impact on the flat ground after rolling over the obstacle is also considered in the theoretical prediction. Theoretical energy loss is easily obtained with the post-obstacle collision velocity ***u***_*p*_ as

$$\Delta E_{RC2}\;=\;\frac{1}{2}u_{p}^{T}M_{C}u_{p},$$ (14)

With ***u***_*p*_ given by (1) using **J**_*R*_ and ***u***_*R*_. Note that the collision matrix **M**_***C***_ in (14) is formed by using a Jacobian for a flat ground contact point, i.e. (4) with the eccentricity *a* = 0. This leads to the total energy loss of the rolling collision

$$\Delta E_{R}\;=\;\Delta E_{RC1}+\Delta E_{RC2}.$$ (15)

### Trivial hopping strategy

For the trivial hopping strategy of the centred wheel, we apply a vertical impulse just before the obstacle, such that the wheel hops over the obstacle. The energy required for this strategy is equal to the potential energy to lift the wheel onto the obstacle

$$\Delta E_{HW}\;=\;mgh.$$ (16)

This energy is exactly lost during ground collision, so that the post-obstacle speed is identical to the initial speed. As in the case of the rotational hopping strategy, the wheel is placed at take-off such that it will hop over the obstacle at apex point. We do not model obstacle overlap due to this placement in the case of slow locomotion speeds.

### Allometric scaling laws

The relation between hip height in running and hopping mammals and birds has been established using biological data [19–21]. The relation for radius to mass was found using linear regression in the logarithmic scales of mass and hip height. The residual least squares solution is

$$R\;=\;0.2063\cdot m^{0.3720},$$ (17)

with a coefficient of determination *r*^2^ = 0.821. The result closely corresponds to findings for quadrupeds in [22]. We therefore employ the radius of gyration and leg mass found in [22] to deduce the moment of inertia of the leg around the hip joint.

The radius of gyration is

$$R_{gy}\;=\;0.047\cdot m^{0.33},$$ (18)

and the leg mass

$$m_{L}\;=\;0.107\cdot m^{1.03},$$ (19)

which leads to a moment of inertia of one leg with respect to pivot point

$$I\;=\;2m_{L}\;R_{gy}^{2}\;=\;0.0004726\cdot m^{1.69}.$$ (20)

The factor 2 accounts for two legs swinging in the same direction as in the case of quadrupedal animals, from which the data was retrieved.

### Experimental set-up

**Fig 3:**
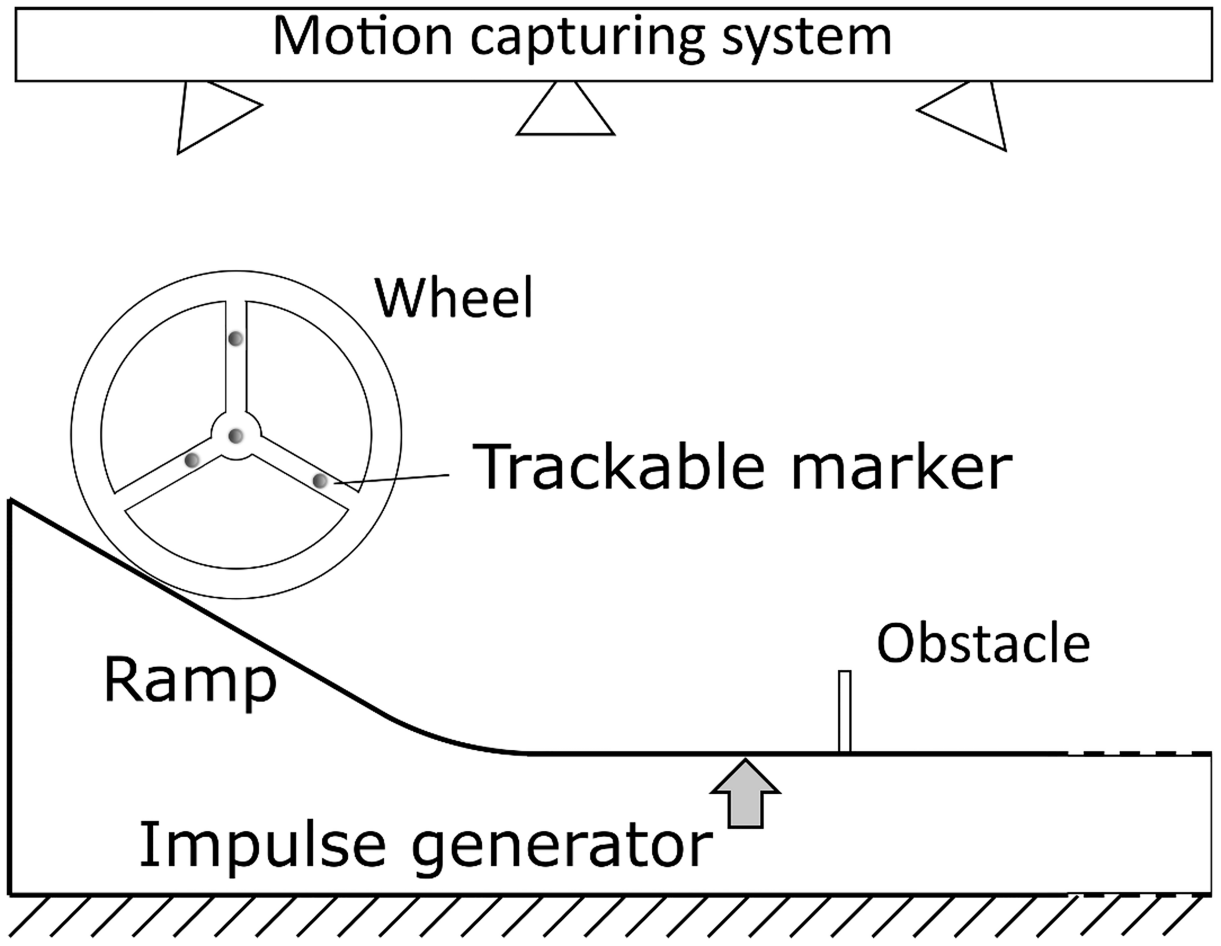
Experimental set-up. Sketch of the experimental conditions. A wooden and rigid wheel-axle system is placed on top of a ramp, released, and guided towards an obstacle. Depending on the locomotion strategy, the system is either passively negotiating the obstacle or hopping over it by an impulse. The motion is recorded with a motion capturing system using four trackable markers placed on one face of the wheel.

The model was tested using a simple experimental setup as is illustrated in Fig 3. The wheel consists of two wooden discs with a radius of *R* = 0.2*m* and 5*mm* thickness which were rigidly connected with an 230*x*13*mm* aluminium rod in the centre of the wheel. A payload was added on the centre of the rod to improve the mass to moment of inertia ratio, resulting in a mass of *m* = 1.417*kg* and a moment of inertia of *I* = 0.008*kg m*^2^. To realize the off-centred mass, the payload was transferred to a second rod placed at a distance of $R/\sqrt{2}$ from the centre of the wheel. This shifted the overall centre of mass roughly to $R/\sqrt{8}$ from the centre of the wheel. The modification led to a change in mass and inertia of *m*_*l*_ = 1.495*kg* and *I*_*l*_ = 0.01*kg m*^2^, respectively. Four reflective markers were placed on the outer face of one disc for the purposes of tracking the wheel motion. The wheel was then placed on one of four different heights on an aluminium ramp and released, which would accelerate the wheel to four pre-impact velocities before reaching a flat wooden track leading to the obstacle. Rubber sheets were fixed to the wooden flat track and metal ramp to guarantee the no-slip condition. The obstacle consisted of wood of 6*mm* thickness and was covered with a thin rubber sheet to reduce slip between the wheel and obstacle. Two obstacle heights (3.6*cm* ≈ 0.2*R and* 7.8*cm* ≈ 0.4*R*) were investigated. After the obstacle, the flat wooden track covered with rubber strips continued to provide space for the collision after the obstacle was surpassed. For the hopping strategy, an impulse generator was installed, which consisted of a lever under the wooden track before the obstacle which would push against the track from below and lift a part of the track. To activate the lever, a weight of 1.5kg was dropped on the far end from a height no higher than the initial ramp position of the system. The dropping height was optimised manually until the required energy was achieved to overcome the obstacle at the tested speed. To track the motion of the wheel and to get an indication of the obstacle position an OptiTrack motion capturing system was used with 12 cameras placed around the testbed. Position data was tracked at a rate of 250Hz with a precision of around 1.5mm and velocities were derived numerically from position data. Pre-collision, flight phase, and post-collision states were obtained from the raw data to retrieve energy conditions and losses. Each pre-collision velocity—obstacle height combination was repeated until five successful samples were recorded.

In the experimental validation of the rotational hopping strategy, we only verify the ground impact energy loss, not the impulsive event at take-off. This is because the ground impact energy loss may be distorted by effects of ground-wheel interactions such as friction, which is not the case in the impulsive take-off event. We test the ground collision loss by throwing the wheel by hand and induce a retracting motion to overcome the obstacle. The energy loss over the impulsive event is then compared to the theoretical prediction according to Eq (2), given the pre-touchdown velocity and touchdown angle. The position during flight phase, collision, and rollout phase were again tracked using the motion capturing cameras.

## Results

### Theoretical results of the rotational hopping strategy

As shown in the methods section, hopping with the rotational hopping strategy as per Fig 2 comes with two energetic costs of different nature. The first cost is associated with the energy required to induce a retracting motion of the wheel according to Eq (7). This cost increases with shorter flight phase time, as the required rotation velocity scales with the flight phase time. The second cost is due to the collision with the ground and is defined by the discrepancy of the kinetic energy before take-off and after collision. The sum of these two costs is the energy required to surpass the obstacle and reaccelerate the body to its initial state before take-off.

**Fig 4:**
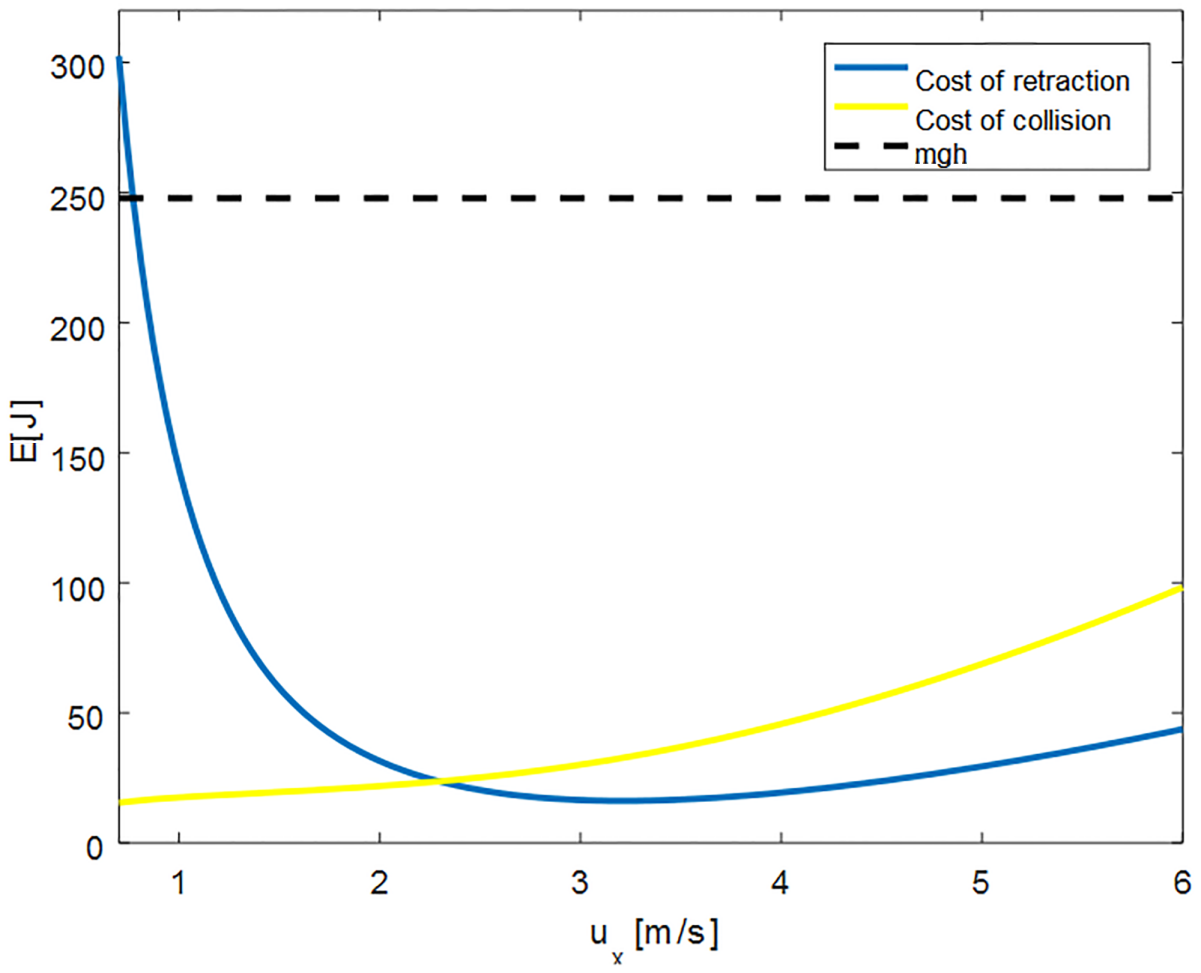
Costs for rotational hopping strategy. The two terms of the energetic collision loss in Eq (9) as a function of forward speed. The blue line corresponds to the cost of retraction or rotation at take-off, and the yellow line corresponds to the energetic discrepancy between initial (pre-impulse) and end (post-impact) state. The black dashed line indicates the trivial hopping strategy’s collision loss, equal to *mgh*. The parameters are: mass *m* = 80*kg*, obstacle height of *h* = 0.3*R*, Radius *R* = 1.05*m*, moment of inertia *I* = 0.78*kgm*^2^, and the take-off angle ϕ* = −0.43*rad*.

Fig 4 shows the two energetic costs of the hopping locomotion of an off-centred wheel as a function of locomotion speed. The wheel properties are defined for a mass of *m* = 80*kg* and an obstacle height of *h* = 0.3*R*, Radius *R* = 1.05*m*, moment of inertia *I* = 0.78*kgm*^2^, and the take-off angle which was found to be ϕ* = −0.43*rad* by solving the implicit Eq (11). We see for the cost of retraction that a high cost occurs for low locomotion speeds, which is explained by the short flight phase time and take-off angle, increasing the first term of Eq (7). For higher speeds, this term becomes smaller, but the second term which accounts for the energy loss due to the required rolling-deceleration increases quadratically with locomotion speed. The presence of these two terms causes a minimum for the cost of retraction, slightly above 3*m*/*s*. For the cost of collision, which we define as the difference between the initial kinetic energy before take-off and the kinetic energy after collision, we see an increase towards higher locomotion speeds, which is explained by the dependency of collision loss with the quadratic form Δ*E* = *u*^−^Φ*u*^−^/2 in Eq (2). The collision loss is significantly lower in the displayed range than for the case of a wheel with centred mass which requires the potential energy *mgh* to overcome the obstacle. This is due to the off-centred position of the wheel and can be comprehended as follows: If the moment of inertia around the centre of mass was zero in the off-centred wheel, we could find a landing angle which causes no energy loss during collision. This angle is such that the touchdown velocity of the centre of mass is perpendicular to the vector pointing from centre of mass to ground contact point. In the case of systems with smaller moments of inertia as compared to their masses, this effect can reduce the collision losses as compared to a wheel with centred mass.

Fig 4 shows that for speeds higher than 3*m*/*s*, the rotational hopping strategy predicts a cost of retraction of roughly 1/3 of the energetic cost of collision, and collisional energy loss is 2/3. In accordance with these findings, studies of running guinea fowls report swing leg costs of 26% of the total energy used for locomotion irrespective of locomotion speed [23], and a study for walking in humans predicts a cost of roughly 30% [24].

### Mass-independent theoretical results

**Fig 5:**
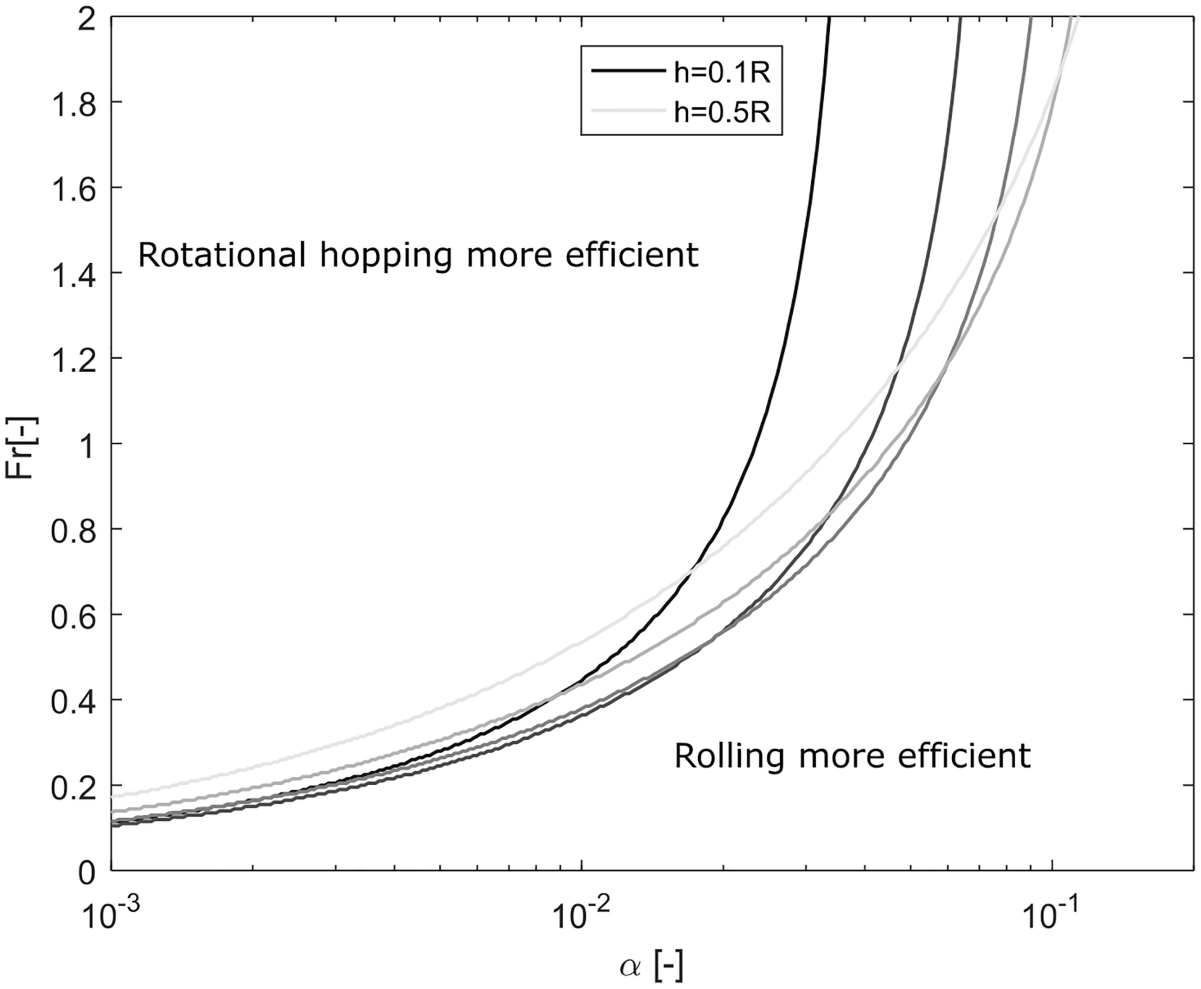
Optimality regions of rotational hopping strategy and rolling strategy. Regions of strategies with least energy loss to overcome an obstacle of height *h* as a function of the moment of inertia factor *α* = *I*/*mR*^2^, and the Froude number $Fr\;=\;u_{x}^{2}/(gR)\;$. The results shown are independent of the mass of the wheel.

The rotational hopping strategy depends on a low moment of inertia to exploit its advantages over other strategies. Surprisingly, we find that only if the moment of inertia scales with *I* = *αmR*^2^, i.e. the square of the radius, where we refer to the mass independent quantity *α* ≠ *α*(*m*) as the moment of inertia factor, the ratio of energy loss of rolling and hopping is independent of the mass, and solely depending on the Froude number $Fr\;=\;u_{x}^{2}/(gR)\;$ for legged locomotion (which is the square of the Froude number used in continuum mechanics) and the obstacle height *h*. Fig 5 shows regions of optimality as a function of the factor *α*, the Froude number, and obstacle heights between *h* = 0.1*R* and *h* = 0.5*R*. We see that hopping is more efficient for higher Froude numbers and low values of *α*, while rolling dominates at low Froude numbers and a larger moment of inertia. The relative improvement of rotational hopping for higher Froude numbers and low moment of inertia factors is explained by the different costs associated with overcoming the obstacle. While the rolling strategy loses kinetic energy when colliding with the obstacle, the rotational hopping strategy has no costs associated with obstacle collisions. On the other hand, while the rotational hopping strategy requires the rotation of the wheel to be reversed to hop over the obstacle, the rolling strategy passively rolls over it. The energetic costs in the strategies scale differently as a function of Froude number and moment of inertia factor, which results in the depicted transition lines These lines represent the section at which rolling and rotational hopping strategy have an equal energetic cost to overcome the obstacle. The effect of obstacle height on the optimal Froude number is more prominent for larger values of *α*, and appears to lose significance for lower values. The optimality transitions as a function of *h* also change their shape first in favour of the rotational hopping strategies for *h* = 0.1*R* to 0.2*R*, and then in favour of the rolling strategy from *h* = 0.3*R* to 0.5*R*.

Note that Fig 5 provides results independent of wheel mass. It thus defines, depending on the wheel properties, whether it is better to roll or to hop over an obstacle of height *h* with locomotion velocity *u*_*x*_.

### Animal-related theoretical results

Before we present the animal-related results in this section, the similarities of our model and legged animal locomotion need to be explained as they are not obvious. Legged locomotion is characterised by repetitive stance and possibly flight phases. The transition from one stride to the next is often accomplished over an impulsive event, in which energy is dissipated in the leg-ground collision. The leg is doing work to compensate for the collisional energy loss (and other losses) during the stance phase. The legs need to be swung back during the gait cycle to allow for a repetitive motion. Our rotational hopping model accounts for all the above as follows: flight and stance phase are both modelled; collisional energy loss is modelled at flight-stance transition through inelastic collisions; energy deficit at the end of the cycle accounts for the work that needs to be done to reaccelerate to the initial wheel state; rotational motion is induced during flight to reset wheel posture. For the rolling strategy, the wheel-obstacle collision is identical to collisions as in the rimless wheel model for walking [5–6], which becomes obvious by drawing spikes from centre of mass to ground contact and obstacle contact in the rolling strategy obstacle collision. There are, of course, limitations of our simple collisional models to capture the complex energetics of legged locomotion. Many effects add to the complexity of legged locomotion, e.g. terrain properties, leg morphology and compliance, friction with the ground, etc., which are not captured by our simple model. The results presented in this section assume that legged animals are underlying the same physical laws of overcoming an obstacle as our wheel model and need to be interpreted with the above limitations in mind.

We now study the energetic costs for the three strategies to overcome an obstacle between *h* = 0.1*R* and *h* = 0.5*R*. We assign animal properties to the wheel, by setting *R* to the animal leg length, *m* to its body mass, and *I* to the leg moment of inertia around the hip. The exact scaling laws are presented in the methods section. As the moment of inertia does not scale proportionally to the square of the radius as follows from (20) (radius of gyration scales differently from leg length), the results now depend not only on the locomotion speed, but also on the mass. The energetics of the respective strategies are computed as described in the method section, with (15) for rolling, (16) for trivial hopping, and (9) for rotational hopping.

**Fig 6:**
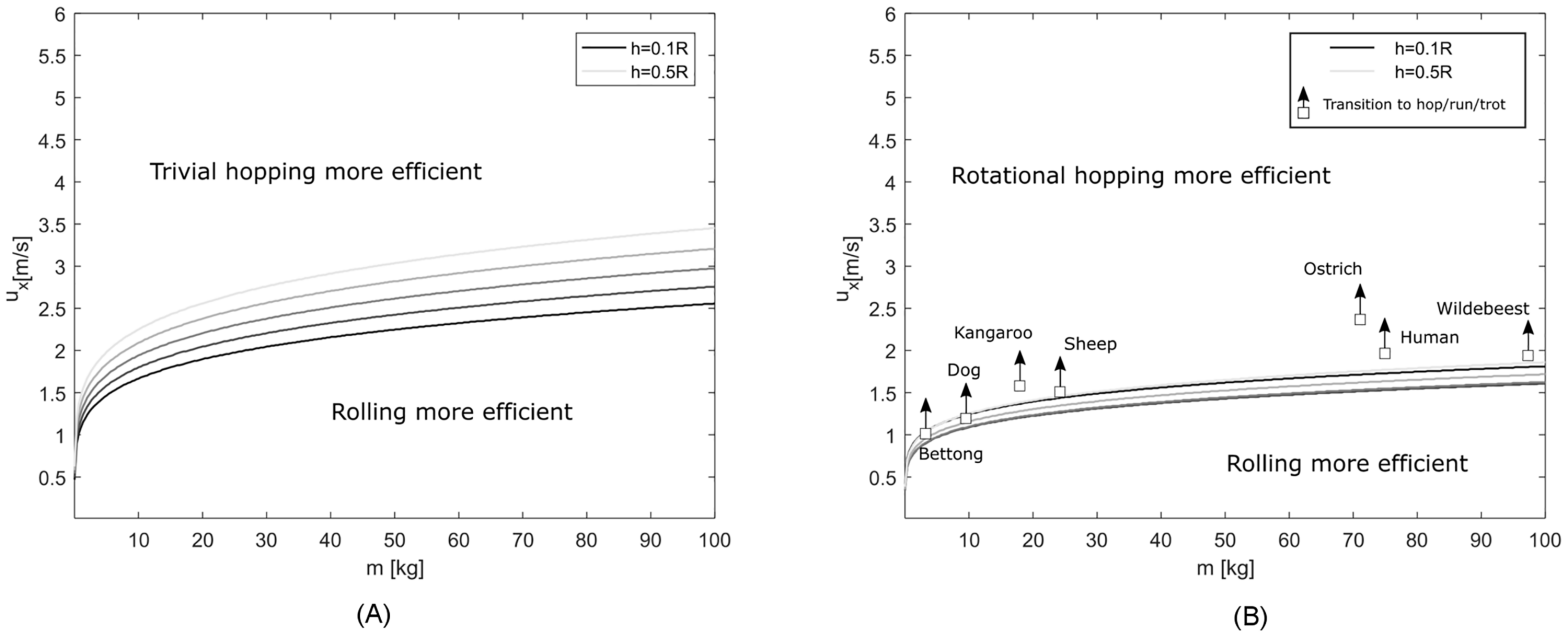
Optimal locomotion strategy for animal related parameters. Regions of optimal strategies as a function of body mass *m*, locomotion speed *u*_*x*_, and obstacle height *h*. Parameters are set using the allometric relations (17)–(20), which scale the wheel radius *R* such that it corresponds to the animal leg length, the point mass *m* corresponds to animal body mass, and the moment of inertia around the centre of mass *I* corresponds to leg moment of inertia around the hip. (A) Rolling strategy compared to trivial hopping strategy and their optimal regions. (B) Rolling and rotational hopping strategy and their optimal regions. Walk to hop/run/trot gait transitions for various animals are indicated [26–30].

Fig 6A shows regions of optimal strategy as a function of body mass, locomotion speed, and obstacle height for the trivial hopping and rolling in terms of energy required to overcome an obstacle. We see that rolling dominates lower speeds and performs better at higher body masses. The lower the obstacle height, the better the hopping strategy performs. Indeed, we can study the limit case for which the obstacle height vanishes and find that optimality transition from rolling to hopping occurs at a Froude number of 1, with $Fr\;=\;u_{x}^{2}/(gR)$. A simulation study of gait transitions with energy optimality objective [25] reached a similar conclusion, stating that running is preferred over walking for vanishing step lengths at a Froude number of larger than one. Vanishing step lengths would correspond to infinitesimal obstacle heights in our model.

Fig 6B shows the rotational hopping strategy and its optimality transitions from rolling to hopping for the same obstacle heights and mass/locomotion speed as in 6A. We see that even though rolling is still superior to hopping for low locomotion speeds, the rotational hopping strategy takes over optimality at lower speeds than in the trivial hopping case. Fig 4 already hinted toward this observation, by the rotational strategy’s lower energetic losses. We would like to point out that the improved performance is dependent on our allometric scaling law of moment of inertia with body mass *m*. As we have seen in Fig 5, the optimality transitions are independent of mass if the moment of inertia factor *α* is of the form *I*/*mR*^2^. Since this is not the case in the allometric relations (17)–(20), this is not exactly true. We find, however, that for the allometric scaling law of leg moment of inertia (20), the optimality transition happens above a Froude number of *Fr* = 0.3, only slightly varying with body mass. This is in close correspondence with experimental findings in quadrupedal animals [26]. Note that Fig 6B shows the transition of the best strategy, not the quantitative energetic loss. We found that 0.5m/s away from the transition line, the rolling and hopping strategies roughly differ by a factor of 2 for all masses, which indicates a rather quick change in optimality as a function of locomotion speed. The energy loss difference between hopping and rolling strategies varies less strongly as a function of mass.

As we scaled the moment of inertia of the wheel with the moment of inertia of animal’s legs, we indicated transition speeds from walking gaits to hopping, running, or trotting for various animals, and we observed that they seem to follow the transition lines.

### Experimental results

**Fig 7:**
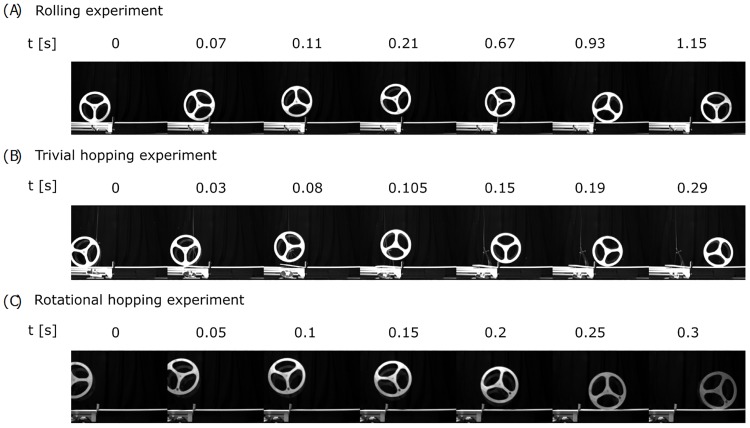
Motion progression of experimentally tested strategies. (A) motion progression of the rolling experiment at an approach speed of 1.8 m/s. (B) motion progression of the trivial hopping experiment at an approach speed of 1.9 m/s. (C) motion progression of the rotational hopping experiment by throwing the wheel. Approach speed corresponds to 2 m/s.

For validation of our model assumptions, we constructed an experimental test platform as shown in Fig 3 and explained in detail in the methods section. Fig 7 shows the motion progression for the three locomotion strategies in experiment. We would like to refer the reader to our supplementary material S1 Video, where we added a slow-motion video of the experimental procedures.

**Fig 8:**
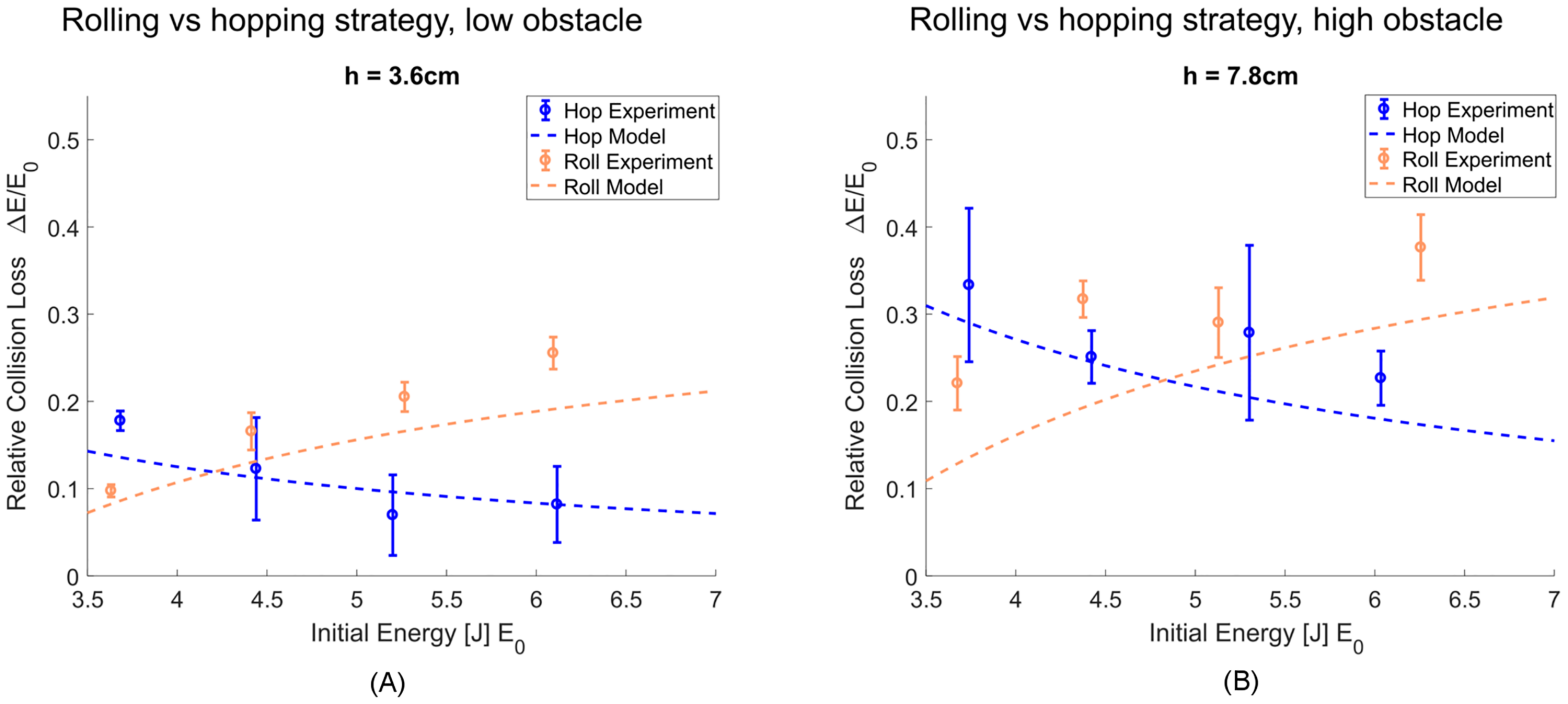
Experimental collision loss for rolling and trivial hopping strategies. (A) Energy loss of rolling strategy and trivial hopping strategy as a function of pre-collision energy for an obstacle height of 0.18*R*. Error bars indicate one standard deviation. (B) Energy loss of rolling strategy and trivial hopping strategy as a function of pre-collision energy for an obstacle height of 0.39*R*. Error bars indicate one standard deviation.

In the simple case of the centred wheel, we performed a controlled set of experiments to assess the collisional energy losses as explained in detail in the methods section. The results for two different obstacle heights as a function of the kinetic and potential energy before the obstacle collision are shown in Fig 8A and 8B, along with the theoretical predictions of the model. The variance arises mainly due to noise in the measurements and the numerical derivative to obtain system velocity but may also stem from ignored effects such as damping, friction, and the omitted lateral dimension. The impulse generator created variations in hopping heights, which was accounted for in post-processing by subtracting the gap potential energy arising due to the distance between wheel apex height in flight phase and obstacle height. Measurement noise and possible elastic restitution at touchdown may have caused the energy loss to fall under the predicted value in some cases. The results show that optimality transition of both theory and experiment occur at around the same initial energy, i.e. approach speed, which validates our model assumption that collisions are the dominant energetic loss during the process of overcoming the obstacle.

**Fig 9:**
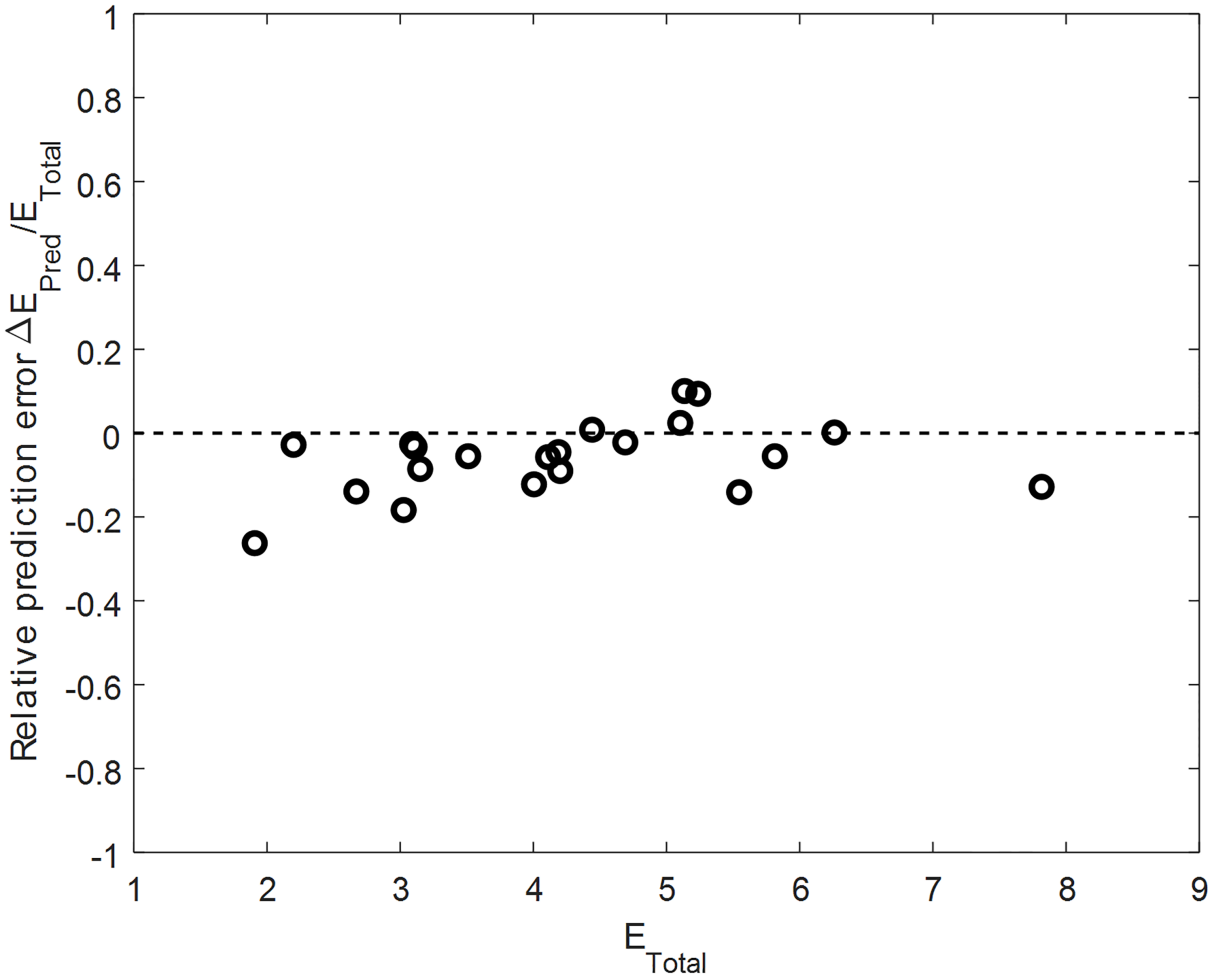
Prediction error of energetic collision loss for rotational hopping strategy. The off-centred mass wheel was thrown over the obstacle, and the touchdown position and velocity state was used to predict theoretical loss Δ*E*_*Theor*_, which was compared to the experimental loss Δ*E*_*Exp*_ to give the prediction error Δ*E*_*Pred*_. The value is normalized with the total energy at touchdown Δ*E*_*Total*_ = Δ*E*_*Kin*_ + Δ*E*_*Pot*_.

For the case of the off-centred wheel, we assessed the accuracy of the energy saving mechanism at collision as predicted by the quadratic form (2). A simple validation is obtained by throwing the wheel to land at different touchdown angles and speeds and comparing the experimental losses with the theoretical prediction. Fig 9 shows the relative error of the predicted energy loss at collision divided by the total energy at touchdown, as a function of the total energy at touchdown. The total energy is the sum of kinetic and potential energy, and the relative error is the theoretical predicted energy loss minus the experimental energy loss. As shown by the results, the experimental losses tend to be larger than the theoretical prediction, which we explain by non-modelled internal losses in the system and friction. The results show that the collisional model of the off-centred wheel can provide accurate predictions of energy loss in experimental conditions. This ensures that the predictions in Fig 4, where we claimed that the off-centred wheel can significantly reduce energetic collision losses as compared to the case of the centred wheel, are valid.

## Discussion

In this work, we have studied the task of a wheel overcoming an obstacle. Because wheels are generally studied in the context of rolling, we aimed to find out if strategies like hopping can be energetically advantageous in this context. Based on collisional mechanics, we have analysed three strategies, namely the rolling strategy, the trivial hopping strategy, and the rotational hopping strategy. Collision based models which study impulsive transitions of the centre of mass [7–10], are powerful tools to understand the underlying physical principles of locomotion, but more complex models might explain effects which are not covered by the simple representation [31]. In this spirit, the inclusion of mass distribution represented by the moment of inertia around the centre of mass, as presented here, was a necessary model extension to find the rotational hopping strategy. Due to this enhancement of the model, we found that the trivial hopping strategy (which is unaffected by the moment of inertia) is not necessarily the most energy-effective in the task of overcoming an obstacle, but, as shown in Figs 4 and 6, that the rotational hopping strategy is superior over a wide range of parameter values. We provided quantitative results that show at which point a wheel better hops than rolls over an obstacle as a function of the Froude number, of the ratio *α* = *I*/(*mR*^2^), and of the obstacle height. The three tested locomotion strategies underlie different sources of energetic cost. The passive rolling strategy collides with the obstacle, the trivial hopping strategy requires the fixed energy *mgh* to be lifted over the obstacle, and the rotational hopping strategy uses a backward rotation to retract its boundary to clear the obstacle and has a reduced collision at touchdown due to the eccentricity of the wheel. Although the strategies are different in their source of energetic loss, a comparison of the collisional losses reveals that the advantage of hopping over rolling as shown in Fig 5 is independent of mass *m*, and can distinctly be determined by only the Froude number and moment of inertia factor for a given obstacle height *h*. The energetics of the three strategies therefore uniquely determine the best way to overcome an obstacle given wheel size, speed, mass distribution, and obstacle height.

The results shown in Fig 6 presented velocity regions of advantages for hopping over rolling strategies for a wheel with the same size, mass, and mass distribution as hopping and trotting animals have. The results show that animal gait transitions from walking to hopping locomotion occur around the boundary from advantages in hopping to rolling. These results point to similarities between the wheel roll-hop transition and gait transitions in legged animals.

One aspect which relates to both systems is the cost to retract the wheel or leg. During flight phase of the wheel model, as seen in Fig 4, the cost to induce the retracting motion at higher locomotion speeds is around 30%. Similar findings were presented for the energetic cost of swing leg retraction in animals [32, 33], and results obtained with mechanical models as in [15, 16] further highlight the importance of modelling swing leg retraction in legged locomotion energetics. Furthermore, the rotational hopping strategy also allows for a redirection of centre of mass after touchdown, which costs no energy due to the smooth rolling transition of the wheel. This effectively results in a trajectory like that in spring-mass models for locomotion [34]. As indicated in [5] and [6], walking energetics can be modelled with a rimless spoked wheel, where the legs are interpreted as spokes. The rolling collision loss studied here is indeed identical to the spoked wheel collision, as revealed by a simple rotation of the system (the effect of gravity is negligible over a collisional event). If walking therefore is associated with the same collisional energy loss as rolling, Fig 6B may explain why animals change their gaits at the transition where hopping becomes more advantageous than rolling. Note that this is only true, however, if the rotational hopping strategy presented in this work does capture the correct energetic costs as found in legged animals. If so, the leg, through walking, may incorporate advantages of rolling at slow speeds, and of hopping at faster speeds. Based on the premise of environments with obstacles and the objective to minimise energy, the wide use of legged hopping rather than rolling in nature seems reasonable.

Our results need to be interpreted with consideration of the model assumptions. A simple obstacle may not completely represent the complexity of natural environments, and legs can certainly exploit more subtle effects than a rigid wheel. Nevertheless, we showed how optimal strategies to overcome an obstacle from rolling to hopping occur in a wheel, and we largely verified the energetic loss predictions in experiments. The results state that if the moment of inertia of the body is small, the superiority of hopping becomes apparent even at low locomotion speeds. The slender legs of animals hint toward a design in nature to reduce leg moment of inertia, possibly to exploit the same physical effects as studied in the wheel model.

## Supporting information

S1 Video Slow motion videos of sample experiments. The three locomotion strategies were filmed with 300fps. Locomotion speeds are around 2m/s and pre-collision energies around 5J. Obstacle height 7.8cm.
(MP4)
